# A Comparison of Methods for Computing the Residual Resistivity Ratio of High-Purity Niobium

**DOI:** 10.6028/jres.116.001

**Published:** 2011-02-01

**Authors:** J. D. Splett, D. F. Vecchia, L. F. Goodrich

**Affiliations:** Statistical Engineering Division, National Institute of Standards and Technology, Boulder, CO 80305-3328; Electromagnetic Division, National Institute of Standards and Technology, Boulder, CO 80305-3328

**Keywords:** cryogenic, electrical resistivity, Kohler’s rule, magnetoresistance, residual resistivity ratio, superconductor

## Abstract

We compare methods for estimating the residual resistivity ratio (*RRR*) of high-purity niobium and investigate the effects of using different functional models. *RRR* is typically defined as the ratio of the electrical resistances measured at 273 K (the ice point) and 4.2 K (the boiling point of helium at standard atmospheric pressure). However, pure niobium is superconducting below about 9.3 K, so the low-temperature resistance is defined as the normal-state (i.e., non-superconducting state) resistance extrapolated to 4.2 K and zero magnetic field. Thus, the estimated value of *RRR* depends significantly on the model used for extrapolation. We examine three models for extrapolation based on temperature versus resistance, two models for extrapolation based on magnetic field versus resistance, and a new model based on the Kohler relationship that can be applied to combined temperature and field data. We also investigate the possibility of re-defining *RRR* so that the quantity is not dependent on extrapolation.

## 1. Introduction

Accurate measurement of the Residual Resistivity Ratio (*RRR*) of niobium samples is important to assure that critical material-purity specifications are met in the construction of superconducting radio-frequency (RF) cavities. The *RRR* value quantifies the overall level of impurities in niobium including: carbon, oxygen, nitrogen, and hydrogen. The *RRR* value also indicates the low-temperature thermal conductivity of the niobium. High-purity niobium, with *RRR* greater than 300, is needed for resonant cavities with the best possible quality factor, *Q*, for particle accelerators in high-energy physics, nuclear physics, light source, and neutron source applications. One possible future application of such a neutron source is to transfer radioactive waste into shorter-lived, less toxic material.

The accepted definition of *RRR* for pure niobium is the ratio of the electrical resistivities or resistances measured at 273 K (the ice point) and 4.2 K (the boiling point of helium at standard atmospheric pressure). However, pure niobium is superconducting below about 9.3 K, so the low-temperature resistance is defined as the normal-state (i.e., non-superconducting state) resistance extrapolated to 4.2 K and zero magnetic field.

**Fig. 1:**
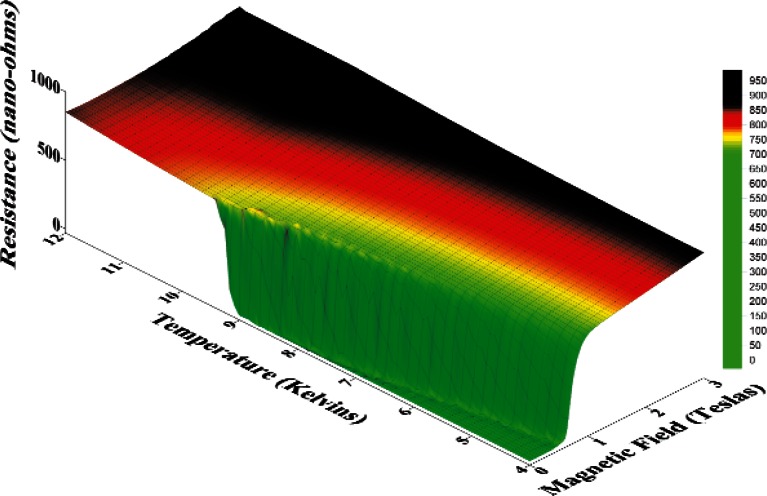
Resistance of a high-purity niobium specimen versus temperature and transverse magnetic field.

A resistance surface as a function of temperature and transverse magnetic field is shown in Fig. 1. When the combination of field and temperature is low enough, the sample is in the superconducting state and the resistance is zero. The transition from normal state to superconducting (the “waterfall” in Fig. 1) occurs at lower magnetic fields as the temperature is increased. For temperatures above 9.4 K or 9.5 K, the sample is normal at zero magnetic field. The surface was generated with measurements of resistance *(R)* versus temperature *(T)* at zero magnetic field and measurements of resistance versus magnetic field *(H)* at various set temperatures.

Three quantities that are common variables in *RRR* measurements of superconductors are temperature, magnetic field, and angle of the magnetic field. In this paper, the term temperature refers to the thermodynamic temperature, *T*, of the specimen in units of kelvins, K. The term magnetic field (or field) refers to the external applied magnetic-field strength, *H.* For convenience and consistency with the practice of the superconductor industry, we express our magnetic field in terms of *μ*_0_*H* in units of teslas, T, where *μ*_0_ = 4π × 10^–7^ H/m, the permeability of free space. The term angle refers to the angle between the magnetic field strength vector and the nominal current direction in the specimen. In this paper, only two angles were used: applied magnetic field parallel and transverse to the specimen current.

The terms resistance (*R*) and resistivity (ρ) are both used in this paper. They are related by

(1) $$R=\frac{\rho \cdot \ell }{A},$$

where *ℓ* is the distance between the voltage taps and *A* is the cross-sectional area of the sample. *R* is the measured, extrinsic parameter of the sample and *ρ* is an intrinsic property of the material. *A* and *ℓ* do change with temperature due to thermal contraction; however, this effect is insignificant for *RRR* measurements. Thus, the ratio of resistances of a sample at different temperatures is the same as the ratio of resistivities. We will typically use resistance when referring to measured values and resistivity when considering theoretical arguments in this paper.

Usually, the *RRR* is derived from either of two methods for obtaining data needed to extrapolate the normal-state resistance of a niobium specimen: (1) measure the normal-state resistance as a function of field at 4.2 K and extrapolate to zero field (field extrapolation), or (2) measure the normal-state resistance as a function of temperature in zero field and extrapolate to 4.2 K (temperature extrapolation). Both methods require the precise measurement of resistance as small as 0.5 μΩ on a specimen that resists wetting by solder. Both methods have their difficulties and each would typically be done with a method-specific experimental apparatus. In the NIST laboratory, however, both types of measurements are made during a single sequence, with one apparatus, to directly compare methods on a given specimen. Because liquid helium boils near 4.0 K at the atmospheric pressure of our test site, data are reported at 4.03 K rather than 4.2 K. We abbreviate 4.03 K as “4 K” throughout this document.

Some laboratories that perform *RRR* measurements using the field extrapolation method use an experimental configuration in which the magnetic field is parallel to the sample current. The NIST system can be adapted to use either a parallel field or a magnetic field transverse to the specimen current. Though values of magnetoresistance depend on field orientation, statistical models we discuss below apply to data from either field orientation. We investigate a new model for estimating *RRR* based on both field and temperature data that take advantage of the Kohler relationship [1, 2].

While it is possible to obtain an estimate for *RRR* for superconducting samples, we need to acknowledge the fact that *RRR* estimates are model-dependent extrapolations used to predict a value that does not exist. We also explore the possibility of changing the definition of *RRR* for superconducting samples so that it is based on actual measurements instead of model-dependent extrapolations. Specifically, we propose defining *RRR* as the ratio of the resistance at 273 K to the resistance at 10 K at zero magnetic field for pure niobium samples.

## 2. Temperature Models

**Fig. 2:**
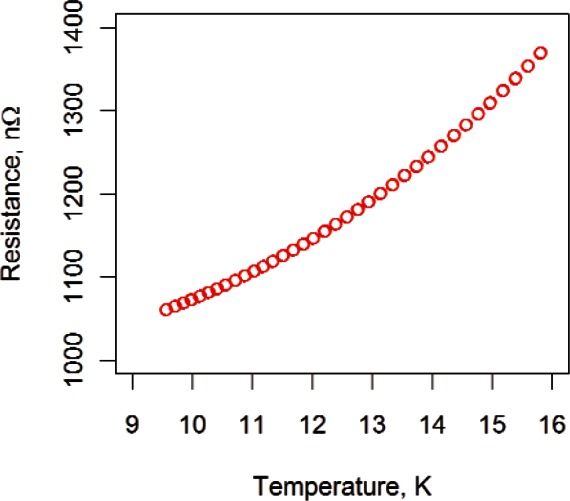
Zero-field resistance versus temperature for sample #2.

**Fig. 3:**
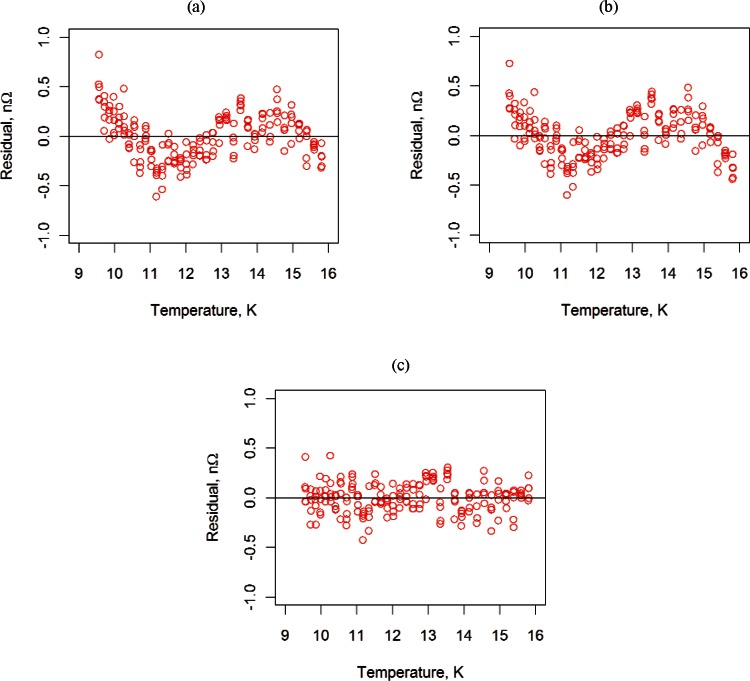
Residuals from three models, (a) T3, (b) TC, and (c) TM, fit to the sample #2 data in Fig. 2.

The first common method used to obtain the extrapolated normal-state resistance is based on the measurement of resistance at various temperatures and zero magnetic field. Three monotonic equations were considered as potential functional models for the resistance (*R*) versus temperature (*T*) relationship:

(2) $$R_{i}=\eta_{0}+\eta_{1}T_{i}^{3},$$

(3) $$R_{i}=\gamma_{0}+\gamma_{1}T_{i}^{\gamma_{2}},$$

(4) $$R_{i}=\phi_{0}+\frac{\phi_{1}}{1+{\left(\frac{T_{i}}{\phi_{2}}\right)}^{\phi_{3}}},\phi_{3}<0,$$

where *η*_0_, *η*_1_, γ_0_, γ_1_, *γ*_2_, *ϕ*_0_, *ϕ*_1_, *ϕ*_2_, and *ϕ*_3_ are unknown parameters to be estimated by linear (or nonlinear) least-squares analysis. We will refer to the three empirical models as the T3 model, the TC model, and the TM (Morgan-Mercer-Flodin) model [3], respectively. Resistance data used for the fits were collected at zero applied magnetic field and increasing values of temperature. We fit measured resistance for temperatures between 9.5 K and 16 K, which is a somewhat conservative range because there may have been some normal-state data below 9.5 K. Figure 2 displays resistance versus temperature data for sample #2, and Fig. 3 displays residuals from fitting the three models.

The estimated values of *γ*_2_ in the TC model Eq. (3) were within a narrow range for samples with typical *RRR* values. For these samples, we obtained a median *γ*_2_ of 3.00, minimum *γ*_2_ of 2.97, and maximum *γ*_2_ of 3.03. (One sample with a very low *RRR* had *γ*_2_ estimated as 3.09.) By comparison, the *γ*_2_ values for aluminum vary between 2 and 5 [4], whereas the *γ*_2_ values for copper vary between 5 and 6, depending on purity [5].

One might think the data shown in Fig. 2 would be fairly easy to fit with a simple function; however, the residuals from the T3 and TC models have a definite pattern, indicating that the models do not account for all structure in the data. One possible explanation for the residual structure is that there is a small systematic error in the thermometer calibration. The TM model appears to fit the data fairly well because the residuals have no discernable pattern. The residuals for all three models are small because most residuals are less than 0.1 % of the measured resistance.

In past interlaboratory comparisons, differences in *RRR* between laboratories have been as large as 10 %, and participating laboratories were required to measure *RRR* with a relative standard uncertainty of less than 5 % of the measured value [6]. Thus, the residuals in Fig. 3, as well as all subsequent residuals, are effectively negligible, and the relative uncertainty associated with all *RRR* measurements reported in this document will be 5 %, which represents a worst-case error.

## 3. Field Models

A second common method of determining normal-state resistance involves measuring resistance over a range of magnetic fields at a fixed temperature, the normal boiling point of liquid helium. In the NIST measurement system, data can be collected for both parallel and transverse magnetic field configurations and for various fixed temperatures from 4 K to 20 K. For a single temperature, we were able to model resistance versus magnetic field for both magnet configurations using the model

(5) $$R_{i}=\lambda_{0}+\lambda_{1}\exp(\lambda_{2}H_{i}^{\lambda_{3}}).$$

We will call this the field model, H. We also fit a Morgan-Mercer-Flodin model, which we refer to as the HM model,

(6) $$R_{i}=\delta_{0}+\frac{\delta_{1}}{1+{\left(\frac{H_{i}}{\delta_{2}}\right)}^{\delta_{3}}},\delta_{3}<0.$$

**Fig. 4:**
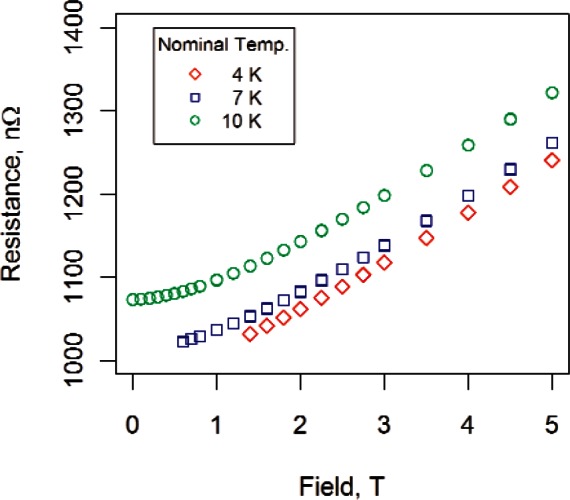
Resistance versus field data for sample #2 for three temperatures and transverse field.

The two empirical, monotonic field models were selected based on fitting the data at 10 K, because the full range of fields could be utilized and the behavior of the curve near zero field could be examined. For data at 7 K we fit fields greater than 0.5 T, and for data at 4 K we fit fields greater than 1.2 T. (Each magnetoresistance curve was examined to determine appropriate limits for the data; some curves required more trimming than others. In general, more data points were trimmed in parallel field than were trimmed in transverse field [1].) These conservative ranges were selected to ensure that all data used in the fit were “normal.” Figure 4 displays measured resistance versus magnetic field for sample #2 for three fixed temperatures and transverse field. Each resistance symbol in Fig. 4 is the result of overlaying 15 repeated measurements at the corresponding combination of field and temperature.

**Fig. 5:**
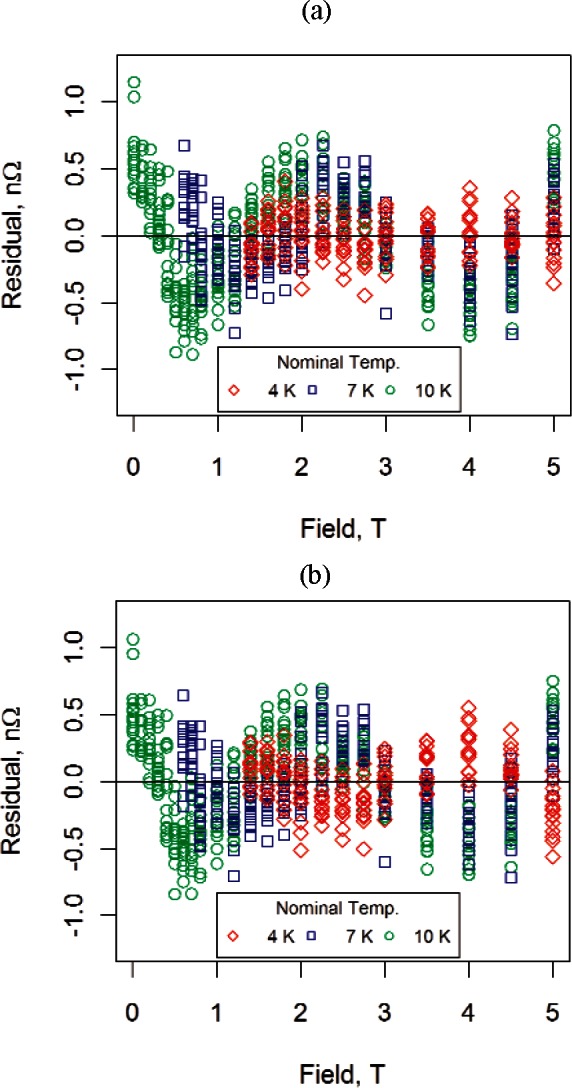
Residuals for sample #2 data (transverse field) for (a) the H model and (b) the HM model. The models were fit to the three temperatures separately. All measured resistance values were greater than 1000 nΩ.

We fit the H model and the HM model to sample #2 data (transverse field) for each temperature separately to obtain the residuals shown in Fig. 5. We display the residuals for all three temperatures on a single graph for comparison. The 4 K residuals for both models are the smallest in magnitude while the 10 K residuals are the largest. Although the residuals themselves are small relative to the magnitude of the measured resistance, the same pattern was obtained for all samples measured, suggesting that the pattern could be an artifact of the measuring system. The most likely source of the residual structure is nonlinearity in the magnet calibration at low fields.

## 4. The Kohler Relationship

**Fig. 6:**
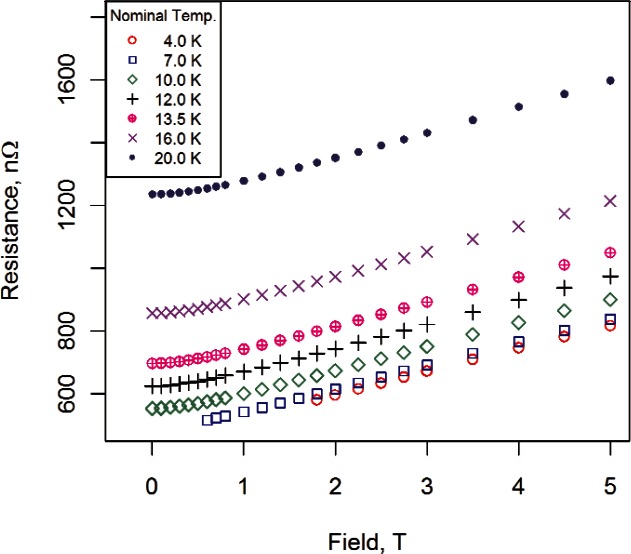
Resistance versus magnetic field at various temperatures for sample #13 using the transverse magnetic field configuration.

We examine a new field-extrapolation measurement method based on the standard 4 K data, but supplemented by additional magnetoresistance data acquired at various fixed temperatures up to 20 K. Figure 6 displays an example of magnetoresistance measurements collected for sample #13 at five normal-state temperatures (10 K, 12.5 K, 13 K, 16 K, 20 K) and two temperatures (4 K, 7 K) that produce the superconducting-to-normal state transition.

The additional magnetoresistance curves at different temperatures, though not needed in traditional extrapolations, have allowed for a potential new measurement procedure derived by generalizing an empirical rule developed by Kohler [2] to describe the behavior of many non-superconducting polycrystalline metals. The mathematical form of Kohler’s rule is

(7) $$\frac{\Delta \rho_{H}}{\rho_{0}}=\frac{\rho_{H}-\rho_{0}}{\rho_{0}}=f\left(\frac{H}{\rho_{0}}\right),$$

where *H* > 0 T is the applied field, *ρ_H_* is the resistivity at field *H*, and *ρ*_0_ is the resistivity at zero field. Kohler’s essential observation was that the function *ƒ* is single-valued over a range of temperatures, increases monotonically, and depends only on the metal and the relative orientation of the field and current. Fickett [5] observed, regarding Kohler’s rule, that “Very few metals show agreement when wide ranges of temperature, purity, defect concentration, and field are used.” For example, copper follows Kohler’s rule, whereas aluminum does not.

**Fig. 7:**
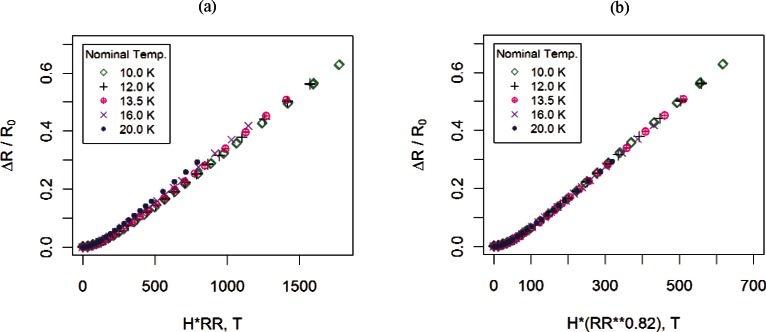
Sample #13 measurements after applying the (a) Kohler transformation and (b) the modified Kohler transformation with *θ*= 0.82. We selected the minimum measured value of resistance as the value of *R*_0_ in the transformations.

A Kohler diagram of resistance data *R* is a plot of Δ*R_H_*(*T*)*/R*_0_(*T*) = [*R_H_*(*T*) − *R*_0_(*T*)]/*R*_0_(*T*) versus *H* ***⋅*** *RR*(*T*), where *T* is the temperature and *RR*(*T*) is the resistivity ratio *R_H_*(273 K)*/R*_0_(*T*). Figure 7(a) displays the Kohler transformation of the normal-state data (*T* ≥ 10 K) shown in Fig. 6.

We developed a generalized version of Kohler’s rule where *RR*(*T*) is replaced by [*R_H_*(273 K)/*R*_0_(*T*)]^θ^, for some constant *θ* to be determined. Figure 7(b) shows a Kohler plot of the data in Fig. 6 after applying a Kohler transformation with 0=0.82. We use this particular value of *θ*, previously determined in [1], for illustration purposes only. (For the data analyzed in this paper, values of *θ* ranged from 0.82 to 1.44 for transverse fields and ranged from 0.72 to 1.12 for parallel fields.) Since *R*_0_ is undefined at the lower temperatures for superconductors, we can only show the data for temperatures where niobium is in the normal state.

The generalized Kohler transformation shown in Fig. 7(b) appears to provide a better alignment of the magnetoresistance curves at different temperatures than the traditional Kohler transformation in Fig. 7(a). The fitting procedure we propose is based on the generalized Kohler transformation and will be called the modified Kohler, or MK model.

In our application, we want to use additional temperature data for the same specimen to impose the general structure at higher temperatures on estimated curves at temperatures where the specimen is superconducting at the lower fields. We use a generalized Kohler transformation to align the temperature data. Absent a theoretical function *ƒ* in the Kohler rule, we have considered various monotonic model functions suggested by Fig. 6. Specifically, we seek a single model that will fit all the individual magnetoresistance curves. For instance, a very good representation of our measurement experiment can be derived from the growth-curve [3],

(8) $$Y_{H}=\beta_{1}{\left[1+{\left(\frac{X_{H}}{\beta_{2}}\right)}^{\beta_{3}}\right]}^{-1},$$

where we define

(9) $$Y_{H}=\frac{R_{H}-R_{0}}{R_{0}},$$

and

(10) $$X_{H}=H{\left(\frac{R_{273}}{R_{0}}\right)}^{\theta }.$$

The value of *R*_0_ estimated from the *T* = 4 K magnetoresistance curve is the parameter needed to compute *RRR*. We re-arrange the growth-curve equation so that the measured resistance is isolated on the left-hand side

(11) $$R_{H}=R_{0}+R_{0}\beta_{1}{\left[1+{\left(\frac{X_{H}}{\beta_{2}}\right)}^{\beta_{3}}\right]}^{-1}.$$

Our goal is to combine magnetoresistance curves and estimate *R_0_* for each curve, as well as the common parameters *θ, β*_1_, *β*_2_, *β*_3_. Since *R*_0_ depends on temperature, a suitable empirical equation for the zero-field temperature response *R*_0_(*T*) can be substituted for *R*_0_ in the model. We use the model

(12) $$R_{0}\left(T\right)=\alpha_{0}+\alpha_{1}{\left[1+{\left(\frac{T}{\alpha_{2}}\right)}^{\alpha_{3}}\right]}^{-1},$$

based on earlier studies on zero-field temperature-extrapolation measurement data. Thus, the modified Kohler model predicts resistance for any given temperature and field.

**Fig. 8:**
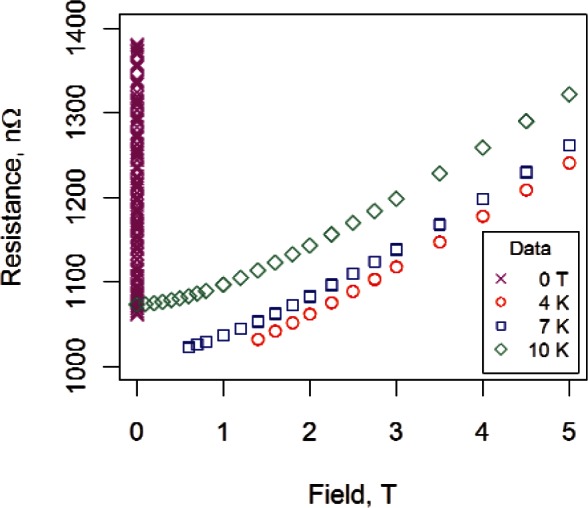
Data used to fit the modified Kohler model for sample #2.

**Fig. 9:**
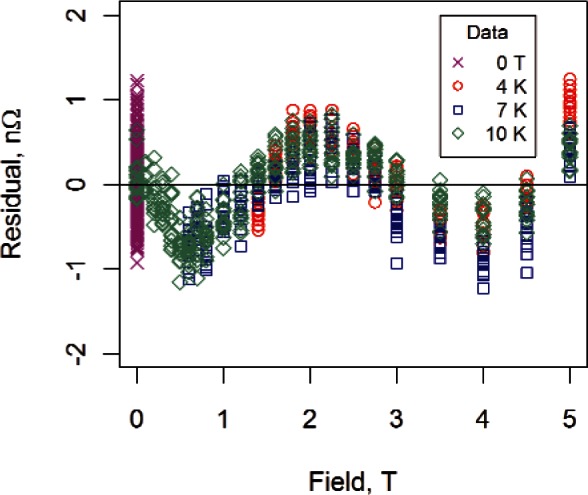
Residuals from modified Kohler fit. All measured resistance values were greater than 1000 nΩ.

Figure 8 shows all the data used to fit the modified Kohler model for sample #2. (We show the 0 T data for completeness.) The zero-field temperature data were constrained to be within 9.5 K and 16 K, the transverse magnetoresistance data were all less than or equal to 5 T, the 7 K transverse magnetoresistance data twere greater than 0.5 T, and the 4 K transverse magneto-resistance data were greater than 1.2 T. Residuals from the fit are shown in Fig. 9. Absolute deviations, all within 0.5 % of measured values of resistance, confirm that the Kohler-based model may offer a promising alternative to other measurement approaches. The residual structure shown in Fig. 9 is similar to the structure in the fit of the magnetoresistance data (Fig. 5).

Our measurement system is capable of producing data over a wide range of temperatures and fields, so we performed an analysis to investigate the sensitivity of the model and the resulting estimates of *RRR* when various subsets of the data were used in the model fit. Table 1 displays *RRR* values computed for sample #13 for various data trimming scenarios.

The results in Table 1 indicate that trimming the magnetoresistance data at 5 T versus 8 T does not appear to have much influence on the value of *RRR* based on the MK model. For example, when the zero-field temperature data and all temperatures for magnetoresistance data are included in the model fit (top two rows in the table), the *RRR* using a maximum of 8 T (413.1) and the *RRR* using a maximum of 5 T (413.4) differ only by 0.3.

The inclusion of zero-field temperature data (for the case where all temperatures for magnetoresistance are included) does seem to have an effect on *RRR*. The *RRR* when zero-field temperature data are included (413.1, 413.4) increases by about 2 when zero-field temperature data are not included (415.6, 415.2).

However, the MK model seems to be the most sensitive to the inclusion of more temperatures for magnetoresistance data (when zero-field temperature data are included) since *RRR* for the case where all temperatures are included (413.1, 413.4) differs by about 3 when only (4 K, 7 K, 10 K) magnetoresistance data are used (416.0, 416.2). There are not enough distinct temperatures to fit the MK model for the case where the zero-field temperature data are excluded and only temperatures (4 K, 7 K, 10 K) magnetoresistance data are used.

For the rest of the paper, the results from the MK model are based on zero-field temperature data (9.5 to 16 K) and magnetoresistance data at 4 K, 7 K, and 10 K. We selected these three temperatures for the magnetoresistance data because that was the minimum common data set for all experimental runs on 14 niobium superconducting samples.

## 5. Model Comparison

At first glance, all six models under consideration are equally plausible for predicting resistance at 4 K and zero field, especially since there is no “true value” we can use for comparison. However, if we assume that the shape of the magnetoresistance curve is similar over the range of temperature from 4 to 10 K, then the MK model has the clear advantage. The MK model imposes the shape of the magnetoresistance curve at higher temperatures (where data are available) on the data at lower temperatures (where data are not available), thus providing a more realistic extrapolation at the lower temperatures than all other models.

**Fig. 10:**
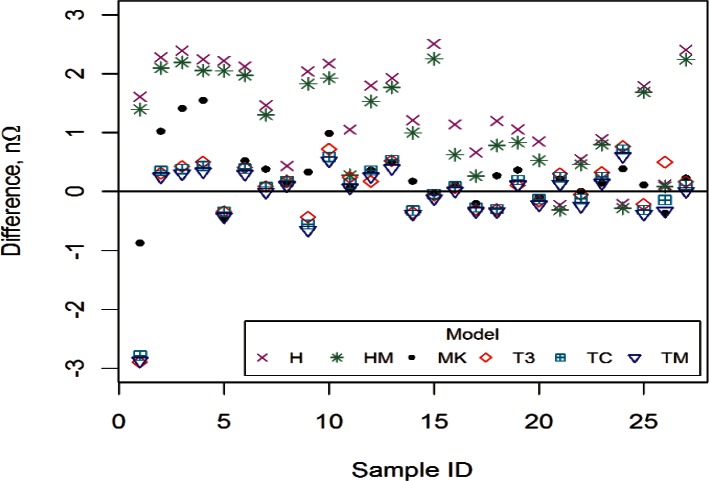
Difference between the mean resistance and the model-based resistance at 10 K and zero field for all six models and all samples.

One way to compare the various models is to plot the differences between the average measured resistance and the model-based values of resistance at 10 K for each sample, as shown in Fig. 10. We would like the measured resistance to be fairly close to the predicted resistance.

The differences for the field models, H and HM, are generally much larger than those for the MK and temperature models, indicating that the field models may be more biased than the other models even when they are fit to the 10 K data where data for zero field exist.

**Fig. 11:**
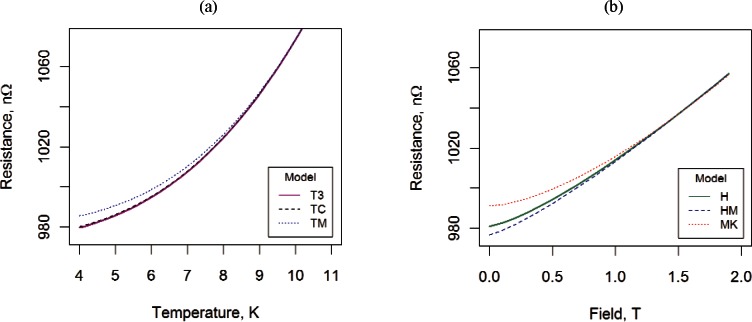
Extrapolated values of resistance for (a) models T3, TC, and TM using the temperature data taken at near zero field and (b) models H, HM, and MK using the transverse field data taken at 4 K.

While Fig. 10 displays a small potential model bias, Fig. 11, which displays predicted values of resistance for both field and temperature models, provides a visual representation of the large effect of model dependence on the extrapolated resistance.

The effect of model dependence on the extrapolation of resistance is also evident in the estimated *RRR* values. Table 2 displays the percent differences between *RRR* based on the MK model (column 2) and *RRR* for each of the other five models (T3, TC, TM, H, and HM) at 4 K and nominally zero field for 14 niobium superconducting samples. Because we assume the MK model provides the best method for extrapolation, we compare each model to the MK model. Samples #1, #2, #10, #11, and #13 were measured on more than one occasion. For consistency, the MK model was fit to the combined magnetoresistance data (4 K, 7 K, and 10 K) and temperature data at zero field for all samples even though some samples have additional magnetoresistance data at higher temperatures.

The *RRR* estimates for the MK model are always lower than the estimates for all other models. The *RRR* values produced by the TM model are the closest to the MK estimates, while the H and HM models produce the largest values of *RRR*. The parallel-field and transverse-field *RRR* estimates are similar for a single sample for the MK model; however the H and HM models can produce wildly different estimates, depending on the magnetic field orientation.

**Fig. 12:**
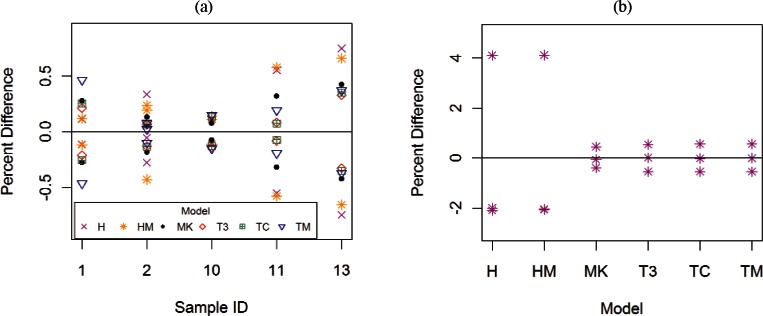
Percent difference between *RRR* estimates and the average *RRR* for (a) each sample (with repeat measurements) and model combination for the transverse field orientation, and (b) sample #13 and each model for the parallel field orientation. These two plots show data that have been normalized for each sample and each model separately.

Figure 12 demonstrates that samples with multiple measurements in transverse field have fairly repeatable values of *RRR* for a given model. Figure 12(a) shows the percent differences between estimated *RRR* values and the average *RRR* value for each sample and model combination for the transverse field orientation. Figure 12(b) shows the percent differences between estimated *RRR* values and the average *RRR* for sample #13 and each model for the parallel field orientation. The percent differences among all samples (with repeat measurements) and estimation methods in transverse field are within 0.8 %. Measurements taken in parallel field for sample #13 are not as repeatable for the H and HM methods since those percent differences are both about 4.1 %.

We think the *RRR* values produced by the MK method are the best possible estimates of *RRR*; however, this method is not very practical for routine characterization. The T3, TC, TM, H, and HM methods (transverse field only) differ from the MK estimate of *RRR* by as much as 2.70 %, 2.39 %, 1.55 %, 3.77 %, and 3.24 %, respectively, indicating that *RRR* estimates in other laboratories may have similar biases if they do not use the MK method. Of course, the bias and repeatability associated with our particular measurement sys-tem may not be typical of other measurement systems.

## 6. A New Definition of *RRR* for Superconductors

Although all the models we consider result in plausible *RRR* values, the quality of the *RRR* estimate depends on the ability of the model to extrapolate beyond the range of data. However, it is difficult to decide which model is best because the value we are trying to predict does not physically exist.

Other superconductors, such as Nb-Ti and Nb_3_Sn wires, use a different definition of *RRR*. The *RRR* of these composite wires is based on the measured low-temperature resistance just above the transition temperature (about 9.3 K for Nb-Ti and 17 K for Nb_3_Sn) [7,8]. Composite superconducting wires incorporate a significant fraction of normal conducting material, such as copper, to improve their thermal stability. For these wires, the *RRR* is an indication of the purity and thermal conductivity of the stabilizer, not of the superconducting component. For composite wires, the *RRR* is a stability figure of merit. The measured composite wire normal-state resistance is not extrapolated to 4.2 K (or the application temperature) mainly because the resulting *RRR* would be nearly the same as the normal-state *RRR* and extrapolation could give incorrect results. Thus, we investigate the possibility of changing the definition of pure niobium *RRR*.

We think a new definition of *RRR* that does not depend on extrapolating an arbitrary model is needed for pure superconducting materials. We propose defining *RRR* to be the ratio of the resistance at 273 K to the resistance at 10 K and zero magnetic field, both of which can be measured. Table 3 lists the percent difference between *RRR* based on measurements and *RRR* based on the six models for each sample at 10 K.

The percent differences for all temperature models are within 0.44 %, and the percent differences for field models are all within 0.49 %. For all samples, the H and HM models produce higher *RRR* values than the *RRR* based on data (column 2). The remaining models produce values that are quite similar to the *RRR*. In general, the percent differences based on actual data at 10 K (Table 3) are smaller than the percent differences for extrapolated values at 4 K (Table 2).

**Fig. 13:**
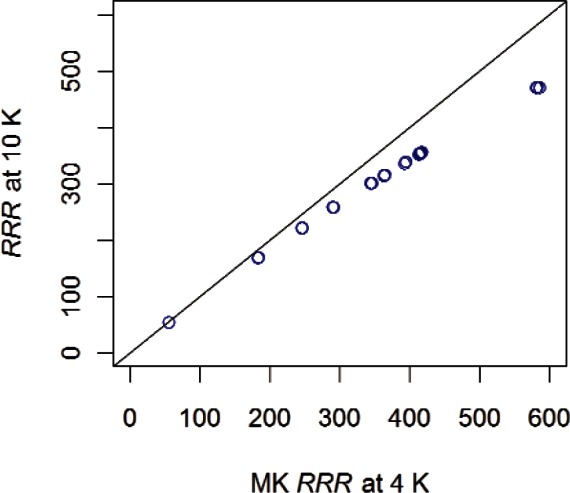
Relationship between estimated *RRR* at 10 K based on data and extrapolated *RRR* at 4 K based on the MK model for 14 samples. A reference line at 45° is shown to indicate perfect agreement between estimates.

Figure 13 displays the estimated *RRR* at 10 K based on data and the extrapolated MK *RRR* at 4 K for 14 samples. Similar patterns were observed for graphs based on the remaining five models. Since the relationship between the *RRR* values at 4 K and 10 K appear to be highly correlated, the new value of *RRR* would have the same meaning as the current value but would just have a different, somewhat distorted scale. For pure niobium, the effect of changing the low-temperature resistance definition from 4.2 K to 10 K changes the *RRR* significantly, especially at higher values of *RRR* which is the region of interest.

**Fig. 14:**
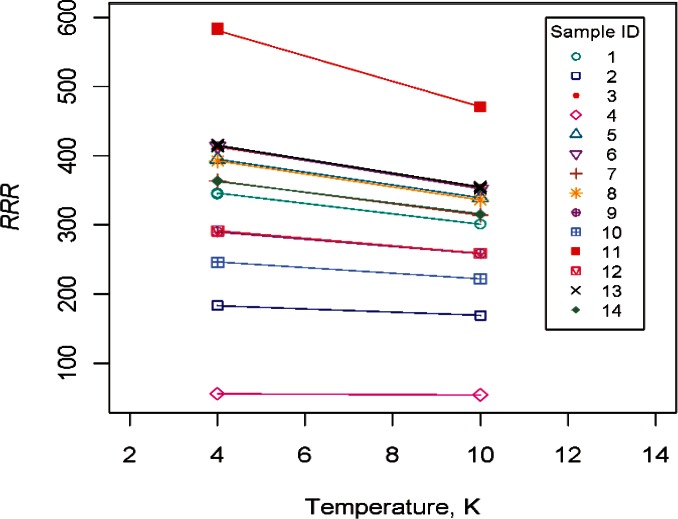
Extrapolated *RRR* at 4 K based on the MK model and estimated *RRR* at 10 K based on data for 14 samples.

Figure 14 displays extrapolated *RRR* values at 4 K based on the MK model and *RRR* values at 10 K based on data for the 14 samples. The relative order of the proposed *RRR* at 10 K is consistent with the extrapolated *RRR* 4 K values. The scaling of the proposed *RRR* at 10 K has the greatest effect on samples with higher *RRR* values.

## 7. A First-Order Correction

Another possible method for computing *RRR* at 4 K is based on a simple empirical relationship that utilizes the difference between the resistance at 10 K and the resistance at 4 K. The measured resistance is an extrinsic material parameter that depends on the voltage tap spacing, cross-sectional area, and the resistivity (an intrinsic material parameter) of the superconductor. Thus, the difference in the low-temperature *R* must be related to an intrinsic parameter to be applied to measurements with different voltage tap spacing and cross-sectional area in general. The temperature dependence of the total resistivity *ρ* (*T*) [9] is

(13) $$\rho \left(T\right)=\rho_{p}\left(T\right)+\rho_{I},$$

where *ρ*_p_(*T*) is the temperature-dependent intrinsic phonon resistivity and *ρ*_I_ is the *temperature-independent* resistivity due to impurities. It is well known that the second term has a small dependence on temperature [9], but we will ignore this for our first order correction. The difference between *R*(10 K) and *R*(4 K) is proportional to the difference between *ρ*_p_(10K) and *ρ*_p_(4K), which in turn is proportional to *ρ*_p_(273 K). The measured R(273 K) is also approximately proportional to *ρ*_p_(273 K), assuming that *ρ*_I_ is much less than *ρ*_p_(273 K). Thus,

(14) R(10K)−R(4K)≈κR(273K),

or

(15) R(4K)≈R(10K)−κR(273K),

where *κ is* a proportionality constant. We estimate *κ by*

(16) $$\kappa \approx \frac{R\left(10K\right)-R\left(4K\right)}{R\left(273K\right)},$$

where *R*(10 K) and *R*(273 K) are based on measurement data and *R*(4 K) is the predicted resistance based on the MK model.

**Fig. 15:**
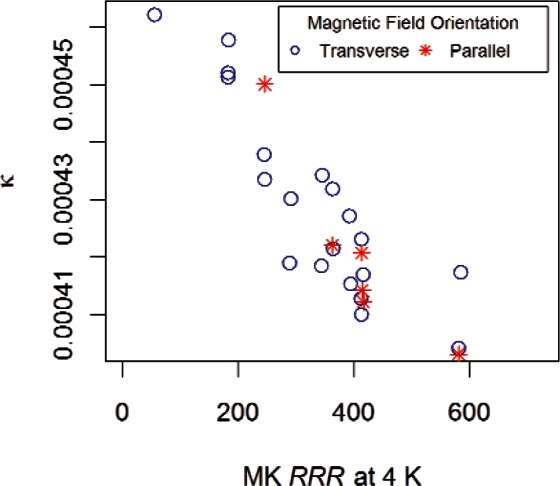
The proportionality constant, *κ*, versus estimated *RRR* at 4 K based on the MK model for all 14 samples.

Figure 15 shows the estimated values of *κ* versus the *RRR* at 4 K using the MK model for 14 samples. The values range from 0.00040 to 0.00047 and vary systematically with *RRR.* Since many materials have slight temperature dependence due to impurities [9], the dependence of *κ* on *RRR* is expected. To provide a single, conservative, proportionality constant for all niobium samples, we recommend using the minimum value of *κ* = 0.000403. This value would be more appropriate for samples having high *RRR* values.

Table 4 lists values of *κ,* estimates of *RRR* at 4 K based on the MK model, *RRR* computed from the minimum value of *κ* (0.000403), and the percent difference between *RRR* based on the MK model and *RRR* values based on the minimum *κ. RRR* values based on the minimum *κ* were computed from measurements of *R*(10 K). The values of *RRR* based on the minimum *κ* are all within 1.16 % of the *RRR* values computed based on the MK model. The percent differences are consistent with those recorded in Table 2 for the various models.

The value of *κ* may depend on the purity of the niobium, and this value may need to be re-determined periodically, as the types and amounts of various impurities change with material source and processing techniques. The proportionality constant corresponding to 4.2 K would be 0.0004.

As mentioned earlier, magnetoresistance curves for temperatures other than 4 K, 7 K, and 10 K were available for some samples. We investigated the effect of fitting all available data on the value of *κ* and found that the minimum *κ* decreased from 0.000403 (based on three temperatures) to 0.000395 (based on all available temperatures). However, values of *RRR* computed when *κ*= 0.000395 differed from the *RRR* reported in Table 4 (column 4) by at most 0.5 % for the highest *RRR* samples.

## 8. Conclusions

Several monotonic models were investigated for fitting resistance versus magnetic field and/or temperature for the purpose of computing the *RRR* of pure niobium samples. Because we assume that magnetoresistance data at 4 K are from the same family of curves as data at 10 K, where the zero-field behavior can be measured directly, we think the MK model provides the best extrapolated resistance values. Thus, we compared five models to the modified Kohler model. While the modified Kohler model was probably the best choice for predicting resistance at 4.2 K or 4.03 K, none of the models can be proven to be correct, because niobium is superconducting at 4.2 K or 4.03 K and zero magnetic field. The TM model also performs well and does not require the large amount of data needed for the MK model.

We propose a new definition of *RRR* for niobium based on measurements at 10 K instead of model-dependent extrapolations to 4.2 K or 4.03 K. The proposed *RRR* has many advantages over the current *RRR*: it is based on measurements, there is no model needed, and there is no need to extrapolate to a point that does not exist for superconducting materials.

Our recommendations are as follows.

1. Redefine *RRR* for niobium based on measurements at 10 K. Although the *RRR* scale would be altered, thus slightly penalizing samples with larger *RRR* values, there would be no bias or uncertainty due to model fitting in the resulting-values. Alternatively, the *RRR* at 4.2 K could be estimated using
  (17) RRR=R(273K)/[R(10K)−0.0004R(273K)],
  This first-order correction removes much of the distortion and penalty of making the *RRR* determination using two measurements, *R*(10 K) and *R*(273 K), and no extrapolation is needed once a value of *κ*(0.0004 based on data presented here) has been established.
2. Although the results are less definitive, resistance versus temperature data at zero field may be used to fit the TM model. This model provides *RRR* estimates that are closest to the MK values for many different samples. The TC model can also be used. The γ_2_ exponent might be useful for indicating problems (noisy data, high resistance or temperature uncertainty) or changes in impurity levels if the exponent deviates significantly from 3.00.
3. If magnetoresistance data are used to calculate *RRR*, we recommend the transverse magnet orientation over the parallel magnet orientation. Although trimming data for the upper fields appears to have little influence on *RRR* estimates, trimming data for the lower fields must be done with care. The lower-field data must be trimmed so that the points do not appear to be decreasing (data points just above the overall transition may not be fully in the normal state). Also, it may be better to have more data points in a narrower field range up to 5 T rather than spread out over a wider range of fields with points above 5 T.

## Figures and Tables

**Table 1:** *RRR* values for sample #13 based on the modified Kohler model for various data trimming scenarios. The designation of “All” temperatures in column three includes 4, 5, 6, 7, 8, 9, 10, 12, 13.5, and 16 K.

Zero-Field Temperature Data Included (9.5 – 16 K)	Maximum Field for Magnetoresistance Data (T)	Temperatures for Magnetoresistance Data (K)	*RRR*
Y	8	All	413.1
Y	5	All	413.4
Y	8	4, 7, 10	416.0
Y	5	4, 7, 10	416.2
N	8	All	415.6
N	5	All	415.2

**Table 2:** Percent differences between *RRR* at 4 K based on the MK model (column 1) and the *RRR* at 4 K extrapolated from each of the remaining five models. The shaded rows indicate results based on parallel-field measurements; all other rows were based on transverse field measurements

Sample	*RRR* MK	T3 (%)	TC (%)	TM (%)	H (%)	HM (%)
1a	344.1	1.69	1.88	0.67	2.89	2.59
1b	346.0	1.56	1.83	1.05	2.55	2.27
2a	183.4	1.10	1.05	0.49	1.37	1.37
2b	183.3	1.19	1.13	0.51	1.05	1.49
2c	182.8	1.23	1.16	0.63	1.06	1.05
2d	183.4	0.98	0.93	0.30	3.34	3.26
3	413.3	2.19	2.01	1.33	2.33	2.31
4	55.7	0.54	0.39	0.17	0.37	0.38
5	394.7	2.03	1.92	0.86	1.99	1.86
6	413.5	1.92	1.99	1.16	3.01	2.91
7	363.3	1.79	1.70	0.80	2.12	1.9
8	392.4	1.81	1.70	0.91	1.14	0.82
9	289.6	1.83	1.82	1.09	1.73	1.64
10a	245.9	1.52	1.50	0.93	1.99	1.95
10b	245.5	1.39	1.38	0.77	1.90	1.88
10c	246.5	1.08	1.09	0.54	17.52	18.04
11a	581.7	2.70	2.39	1.19	7.62	7.57
11b	585.4	2.22	1.89	0.94	5.76	5.65
11c	581.0	2.94	2.67	1.64	34.93	20.21
12	291.2	1.61	1.63	0.79	2.31	2.28
13a	416.2	2.05	2.12	1.44	3.77	3.24
13b	412.6	2.25	2.28	1.55	3.11	2.76
13c	413.4	1.96	2.01	1.31	15.34	11.57
13d	416.9	2.21	2.28	1.58	7.69	4.08
13e	414.8	2.19	2.21	1.53	8.12	4.63
14a	364.1	1.99	2.15	1.35	2.30	1.84
14b	363.2	1.94	1.99	1.21	9.24	6.63

**Table 3:** Percent differences between *RRR* at 10 K (column 2) and the predicted *RRR* at 10 K based on the six models. The shaded rows indicate results based on parallel-field measurements, all other rows were based on transverse field measurements

Sample	*RRR*	MK (%)	T3 (%)	TC (%)	TM (%)	H (%)	HM (%)
1a	300.8	−0.13	−0.44	−0.42	−0.43	0.24	0.21
1b	300.8	0.06	0.03	0.05	0.04	0.27	0.23
2a	169.2	0.02	0.03	0.02	0.01	−0.02	−0.03
2b	169.3	0.00	0.00	−0.01	−0.02	0.05	0.04
2c	168.9	0.01	0.03	0.02	0.01	0.08	0.07
2d	168.9	0.04	0.07	0.07	0.06	−0.02	−0.03
3	353.4	0.02	−0.04	−0.06	−0.07	0.33	0.31
4	54.3	−0.01	0.01	0.00	−0.01	0.00	0.00
5	339.1	0.04	0.03	0.02	0.00	0.41	0.39
6	352.0	0.18	0.06	0.06	0.05	0.41	0.38
7	314.1	0.21	0.07	0.06	0.05	0.36	0.33
8	336.1	0.26	0.09	0.07	0.06	0.38	0.35
9	258.3	−0.06	−0.04	−0.04	−0.05	0.29	0.26
10a	222.2	0.06	0.04	0.04	0.03	0.23	0.22
10b	221.7	0.04	0.01	0.01	0.00	0.16	0.14
10c	221.9	0.01	0.02	0.02	0.01	0.05	0.02
11a	471.0	0.08	−0.10	−0.13	−0.15	0.46	0.41
11b	470.5	0.22	0.16	0.13	0.12	0.49	0.44
11c	470.8	0.02	0.05	0.03	0.02	0.24	0.06
12	258.8	0.06	0.07	0.07	0.05	0.25	0.23
13a	354.6	0.03	−0.06	−0.06	−0.07	0.22	0.18
13b	352.6	0.00	−0.01	−0.01	−0.02	0.45	0.41
13c	352.2	0.02	0.01	0.02	0.00	0.20	0.11
13d	355.8	−0.04	−0.05	−0.05	−0.06	0.12	0.05
13e	354.0	0.05	−0.05	−0.05	−0.06	0.22	0.14
14a	315.7	0.06	0.02	0.03	0.02	0.17	0.13
14b	314.9	−0.01	−0.03	−0.02	−0.03	0.13	0.08

**Table 4:** Values of *κ*, *RRR* at 4 K based on the MK model, the value of *RRR* based on the minimum *κ*(0.000403), and the percent difference between the MK *RRR* and the *RRR* based on the minimum *κ* for each sample. The shaded rows indicate results based on parallel-field measurements; all other rows were based on transverse field measurements

Sample	*κ*	MK *RRR*	Min. *κ* *RRR*	Min.*κ RRR* – MK *RRR* (%)
1a	0.00042	344.1	342.3	−0.52
1b	0.00043	346.0	342.3	−1.07
2a	0.00046	183.4	181.6	−0.99
2b	0.00045	183.3	181.7	−0.89
2c	0.00045	182.8	181.2	−0.87
2d	0.00047	183.4	181.3	−1.16
3	0.00041	413.3	412.1	−0.28
4	0.00046	55.7	55.5	−0.33
5	0.00042	394.7	392.8	−0.48
6	0.00042	413.5	410.1	−0.82
7	0.00043	363.3	359.6	−1.03
8	0.00043	392.4	388.8	−0.93
9	0.00042	289.6	288.3	−0.46
10a	0.00043	245.9	244.1	−0.74
10b	0.00044	245.5	243.4	−0.85
−10c	0.00045	246.5	243.7	−1.14
11a	0.00040	581.7	581.4	−0.06
11b	0.00042	585.4	580.6	−0.83
11c	0.00040	581.0	581.0	0.00
12	0.00043	291.2	289.0	−0.78
13a	0.00042	416.2	413.8	−0.57
13b	0.00041	412.6	411.0	−0.40
13c	0.00042	413.4	410.4	−0.72
13d	0.00041	416.9	415.3	−0.38
13e	0.00041	414.8	412.9	−0.46
14a	0.00042	364.1	361.7	−0.66
14b	0.00042	363.2	360.7	−0.68
